# ﻿New nephridiophagid genera (Fungi, Chytridiomycota) in a mallow beetle and an earwig

**DOI:** 10.3897/mycokeys.100.111298

**Published:** 2023-12-22

**Authors:** Renate Radek, Christian Wurzbacher, Jürgen F. H. Strassert

**Affiliations:** 1 Evolutionary Biology, Institute of Biology, Free University of Berlin, 14195 Berlin, Germany Free University of Berlin Berlin Germany; 2 Chair of Urban Water Systems, Engineering, Technical University of Munich, 85748 Garching, Germany Technical University of Munich München Germany; 3 Evolutionary and Integrative Ecology, Leibniz Institute of Freshwater Ecology and Inland Fisheries, 12587 Berlin, Germany Evolutionary and Integrative Ecology, Leibniz Institute of Freshwater Ecology and Inland Fisheries Berlin Germany

**Keywords:** Chytrids, *Forficulaauricularia*, Malpighian tubules, *Malpighivincopodagricae*, *Nephridiochytriumforficulae*, *Nephridiophaga*, *Podagricamalvae*, phylogeny

## Abstract

Nephridiophagids are unicellular fungi (Chytridiomycota) that infect the Malpighian tubules of insects. Most species have been found in cockroach hosts and belong to the genus *Nephridiophaga*. Three additional genera have been described from beetles and an earwig. Here, we characterise morphologically and molecular phylogenetically the nephridiophagids of the European earwig *Forficulaauricularia* and the mallow beetle *Podagricamalvae*. Their morphology and life cycle stages resemble those of other nephridiophagids, but their rRNA gene sequences support the existence of two additional genera. Whereas the earwig nephridiophagid (*Nephridiochytriumforficulae***gen. nov. et sp. nov.**) forms a sister lineage of the *Nephridiophaga* cluster, the mallow beetle nephridiophagid (*Malpighivincopodagricae***gen. nov. et sp. nov.**) represents the earliest divergent lineage within the nephridiophagids, being sister to all other species. Our results corroborate the hypothesis that different insect groups harbour distinct nephridiophagid lineages.

## ﻿Introduction

The members of Nephridiophagaceae (‘nehridiophagids’) are unicellular entomopathogens, which reproduce in the Malpighian tubules of insects (Woolever 1966; Lange 1993; Radek and Herth 1999; Voigt et al. 2021). During their life cycle, multinucleate vegetative plasmodia divide into oligo- and uninucleate stages or transform to sporogenic plasmodia. Spores develop within the sporogenic plasmodium by delimination of a portion of cytoplasm including a generative nucleus. Degenerated vegetative (“somatic”) nuclei remain in the cytoplasm of the mother plasmodium. The occasional occurrence of bi- and tetranuclear sporoblasts implies the existence of meiosis or sexual stages (Ivanić 1937; Purrini and Rhode 1988; Radek and Herth 1999). Mature spores are mostly flattened oval, have a spore opening at one side and include a single nucleus (Fabel et al. 2000). An infection with nephridiophagids does not kill its host but reduces its fitness, leading to, for example, lessened mobility, reduced fat reserves and fewer progeny (Strassert et al. 2022).

Only recently, the controversial discussion about the systematic position of nephridiophagids has been resolved by molecular phylogenetic analyses, which recognise them as fungi in the phylum Chytridiomycota (Radek et al. 2017; Strassert et al. 2021). Presently, the four genera *Coleospora*, *Nephridiophaga*, *Oryctospora* and *Peltomyces* are included in the family Nephridiophagaceae (Wijayawardene et al. 2018, 2020). While the monospecific genera *Coleospora* and *Oryctospora* are known from beetles only (Gibbs 1959; Purrini and Weiser 1990), the two *Peltomyces* species have been found to infect a different host each (a beetle and an earwig; Léger (1909)). The majority of the hitherto described nephridiophagids (ca. 14 species) are assigned to the genus *Nephridiophaga* (Radek and Herth 1999; Wijayawardene et al. 2018; Strassert et al. 2021; Voigt et al. 2021), which was erected by Ivanić (1937) who described spores in the Malpighian tubules of the honey bee *Apismellifera*. Due to obscurities regarding this assignment, however, a new conserved type was recently defined for *Nephridiophaga* (*Nephridiophagablattellae* from the German cockroach *Blattellagermanica*; Radek et al. (2022)). *Nephridiophaga* species are mostly known from cockroaches and nephridiophagid species isolated from cockroaches have, so far, been found to be phylogenetically closely related to each other (Strassert et al. 2021). Interestingly, the only nephridiophagid that inhabits a non-cockroach host (an earwig) and for which SSU and LSU rRNA gene sequences are publicly available, has recently been shown to branch as sister to nephridiophagids isolated from cockroaches (Strassert et al. 2021).

In this study, we morphologically and molecular phylogenetically characterise the nephridiophagids from the European earwig *Forficulaauricularia* and the mallow beetle *Podagricamalvae*. We show that, despite morphological similarities to *Nephridiophaga* (the genus they had formerly been assigned to), the here formally described taxa branch not only apart from this genus in phylogenetic trees but also from each other, strengthening the hypothesis that different insect groups host own lineages of nephridiophagids.

## ﻿Methods

### ﻿Insects

The insects used for this study were mallow beetles, *Podagricamalvae* (Coleoptera, Chrysomelidae, Galerucinae), collected in Italy (Syracuse) and individuals of the European earwig, *Forficulaauricularia* (Dermaptera, Forficulidae), collected in France (Tours). Detailed information on the collected insects and their infection status is given in Suppl. material 1. Identifications were based on morphological characteristics as well as mitochondrial COII gene sequence similarities.

### ﻿Light microscopy

Beetles and earwigs were dissected in 0.9% sodium chloride (NaCl) solution. Malpighian tubules were extracted and screened under a light microscope for entomopathogens, especially for nephridiophagids. For the production of stained permanent samples, the infected tubules were then smeared on a microscopic slide, air dried, fixed in 100% methanol for 5 min, stained in Giemsa solution (Accustain, Sigma, diluted 1:10) for 30–60 min, washed with tap water, air dried and embedded in Entellan under a cover glass. All light microscopic samples were observed under a Zeiss AxioPhot microscope equipped with a 40× objective.

### ﻿Electron microscopy

Cover glasses removed from positive squash preparations were used for scanning electron microscopy (SEM). They were air dried in order to prevent parasite loss during further preparation steps. The dried cover glasses were mounted on aluminium stubs with double-sided adhesive tape, sputter-coated with gold in a Balzers SCD 40 and observed using a FEI Quanta 200 ESEM.

For transmission electron microscopy (TEM), infected tubules were fixed in 2.5% glutardialdehyde in 0.1 M cacodylate buffer (pH 7.2) and stored in a fridge for several days. The fixed tissue was then washed three times in buffer, post-fixed in 1% osmium tetroxide (OsO_4_) plus 1.5% potassium ferrocyanide (K_3_[Fe(CN)_6_]) for 1.5 h at room temperature, washed three times, dehydrated in a gradient of ethanol and embedded in Spurr’s resin (Spurr 1969). Ultrathin sections were contrasted with saturated aqueous uranyl acetate ([UO_2_(CH_3_COO)_2_ · 2 H_2_O]) for 30 min followed by lead citrate (C_12_H_10_O_14_Pb_3_) (Reynolds 1963) for 5 min. The sections were examined with a Philips CM 120 BioTwin electron microscope.

### ﻿Sample processing for molecular phylogeny

DNA from infected Malpighian tubules was extracted with the DNeasy Plant Mini Kit (Qiagen) following the manufacturer’s instructions. The ribosomal operon sequence spanning the SSU, ITS, 5.8S and partial LSU region was amplified and sequenced as described in Wurzbacher et al. (2019). Briefly, the ribosomal operon was amplified with the primers NS1short and RCA95m, subsequently purified with magnetic beads (AMPure, Beckmann), barcoded and re-purified and subjected to amplicon sequencing on the PacBio RSII sequencer (Pacific Biosciences) in reads of insert mode (ROS) and an error rate filtering of 0.02. The resulting sequences were aligned, clustered and used for consensus sequence generation as described in Wurzbacher et al. (2019). The sequence of the nephridiophagid from *F.auricularia* was obtained in a similar way by Strassert et al. (2021) with the exception that the MinION (Oxford Nanopore Technologies) was used for amplicon sequencing, but the species has not been formally described.

### ﻿Phylogenetic analysis

Analyses were carried out using the high-performance computing infrastructure Zedat at Freie Universität Berlin (Bennett et al. 2020). Sequences of the SSU and LSU rRNA genes from the earwig nephridiophagid and from the mallow beetle nephridiophagid were aligned together with representatives of diverse fungal lineages using MAFFT L-INS-I v. 7.055b (Katoh and Standley 2013) (Suppl. materials 2, 3) and filtered with TRIMAL v. 1.2 (Capella-Gutierrez et al. 2009) using a gap threshold of 0.3 and a similarity threshold of 0.001. The two alignments were then concatenated using SEQKIT v. 0.11.0 (Shen et al. 2016) and a Maximum-Likelihood tree was inferred with IQ-TREE v. 1.6.12 (Nguyen et al. 2015) under the GTR+F+R5 model, which was determined with ModelFinder (Kalyaanamoorthy et al. 2017) employing the TESTNEW option. Branch support was assed using ultrafast bootstrap approximation (Hoang et al. 2018) (UFBOOT2; 1,000 replicates) and SH-like approximate likelihood ratio test (Guindon et al. 2010) (SH-aLRT; 1,000 replicates). PhyloBayes-MPI v. 1.8 (Lartillot et al. 2013) was used for Bayesian analysis with the GTR model and four categories for the discrete gamma distribution (31,000 generations; burn-in 3,100). Convergence of two independent Markov Chain Monte Carlo (MCMC) chains was tested with BPCOMP and confirmed with MaxDiff reaching 0.03.

## ﻿Results

### ﻿Nephridiophagid in *Forficulaauricularia*

**Figure 1.**
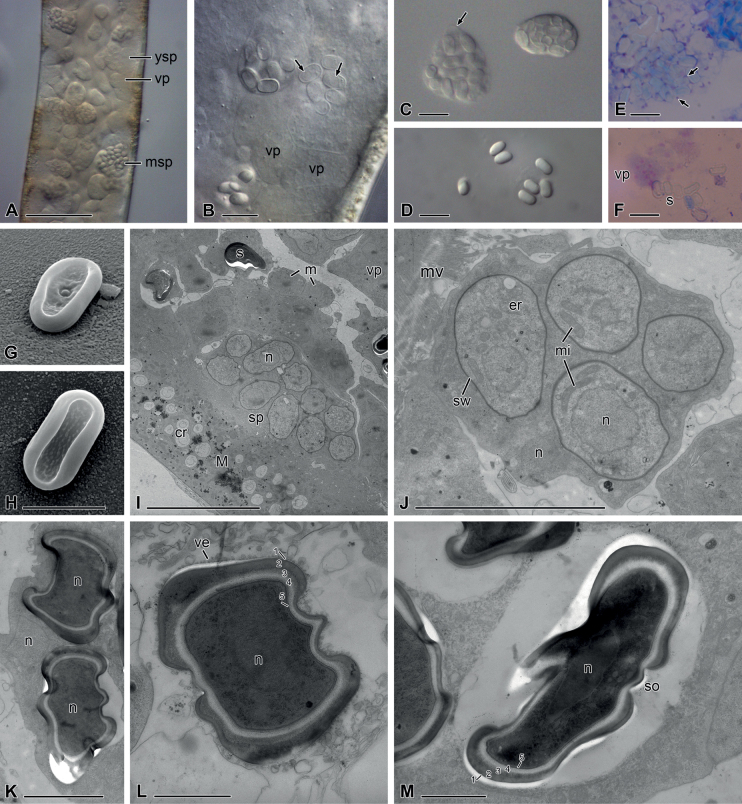
Nephridiophagid (*Nephridiochytriumforficulae*) from *Forficulaauricularia***A–D** differential interference microscopy (DIC) **E, F** Giemsa staining **G, H** scanning electron microscopy (SEM) **I–M** transmission electron microscopy (TEM) **A** Malpighian tubule filled with vegetative plasmodia (vp), young sporogenic plasmodia (ysp) and mature sporogenic plasmodia (msp) **B** young spores have a thin, yet transparent spore wall and a nucleus positioned near a cell pole or centrally **C** left plasmodium with large mature spores, right plasmodium with smaller, younger spores. Arrow points to plasma membrane **D** single mature spores **E** Giemsa staining reveals residual nuclei (arrows) of the plasmodium between mature spores **F** vegetative plasmodium with stained nuclei. s = spores **G, H** flattened oval spores with rim and a central spore opening on one side (**G**) **I** ultrathin section through a Malpighian tubule infected with different stages: small, uninucleate merozoites (m), vegetative plasmodia with several nuclei, sporogenic plasmodia (sp) and mature spores. n = nucleus. The epithelium of the Malpighian tubule (M) contains concretions (cr) **J** young sporogenic plasmodium containing young spores with a thin spore-wall (sw), one nucleus, endoplasmic reticulum (er) and mitochondria (mi) **K** part of a mature sporogenic plasmodium with a residual nucleus and two mature spores with a centrally located nucleus and a thick spore-wall **L** cross-section of a mature spore in the degenerating plasmodial cytoplasm. The spore wall consists of five layers (1–5). ve = vesicles **M** mature spore in longitudinal section showing the thin-walled cap of the spore opening (so). Scale bars: 50 µm (**A**); 10 µm (**B–F, I**); 5 µm (**G, H, J**); 2 µm (**K**); 1 µm (**L, M**).

The lumens of the Malpighian tubules of infected earwigs were filled with different developmental stages (Fig. 1). Sporogenic plasmodia contained 13–37 (mean 19) spores (n = 13; Fig. 1A–C). Giemsa staining revealed the presence of residual vegetative nuclei between the mature spores (Fig. 1D) and a varying number of nuclei in vegetative plasmodia (Fig. 1F). Oval, flattened mature spores measured 5.8–6.9 (mean 6.3) μm in length and 2.9–3.5 (mean 3.2) μm in width (n = 10; Fig. 1E). In scanning electron micrographs, the flattened mature spores revealed a thickened rim and two different sides. One side featured a small, central, rounded spore opening (Fig. 1G), while the other one showed a homogeneous granular surface (Fig. 1H). Different stages of spore formation could be found in ultrathin sections. Regions of future spores were demarcated around single nuclei in the sporogenic plasmodia (Fig. 1I, J). Other nuclei remained in the plasmodial cytoplasm (Fig. 1J, K). Young developing spores revealed an oval, not yet flattened shape, a thin spore wall and nuclei positioned near to a pole or centrally (Fig. 1J). Their cytoplasm had about the same density as that of the mother cytoplasm. Mature spores had a typical oval flattened form and an electron-dense interior with a centrally located nucleus (Fig. 1K–M). Their spore walls were the thickest at the border and thin at the flattened sides and especially thin at the region of the spore opening (Fig. 1L, M). The spore wall was composed of five layers. The outer layer 1 and the inner layer 5 appeared as thin electron-dense lines in the sections (Fig. 1L, M). The other three layers were thicker and became less electron-dense from outside to inside. A layer of small vesicles surrounded the single spores (Fig. 1L).

### ﻿Nephridiophagid in *Podagricamalvae*

**Figure 2.**
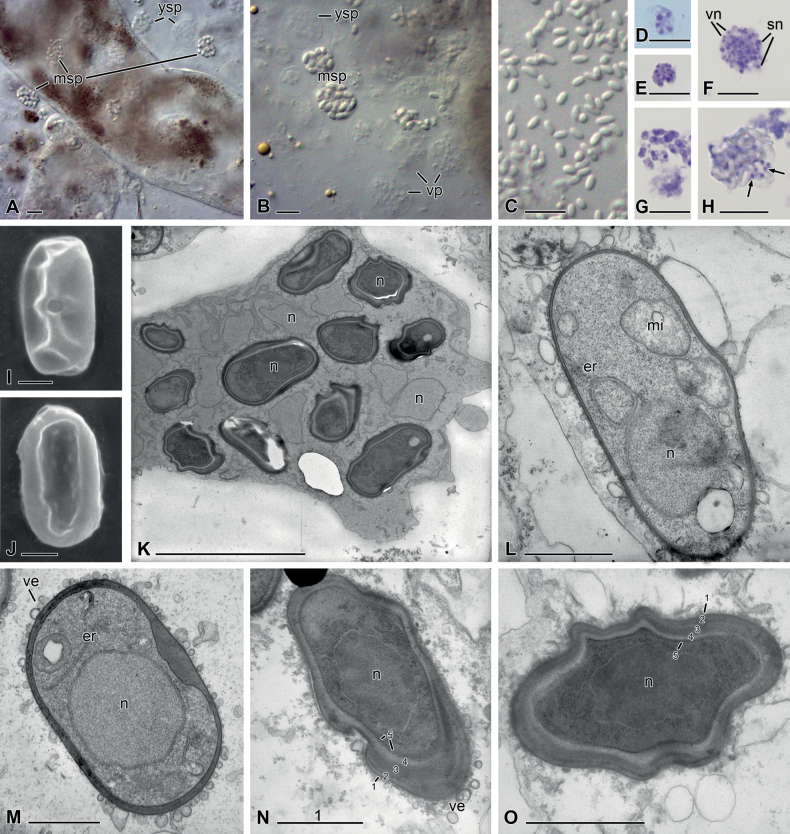
Nephridiophagid (*Malpighivincopodagricae*) from *Podagricamalvae***A–C** differential interference microscopy (DIC) **D–H** Giemsa staining **I, J** scanning electron microscopy (SEM) **K–O** transmission electron microscopy (TEM) **A** fresh squash preparation of infected Malpighian tubules with young sporogenic plasmodia (ysp) and mature sporogenic plasmodia (msp) inside and outside the tubules **B** nuclei of vegetative plasmodia (vp) and young spores in sporogenic plasmodia have less contrast than mature spores **C** mature spores **D, E** vegetative plasmodia with few (**D**) or many (**E**) nuclei **F** young sporogenic plasmodium with small vegetative nuclei (vn) and larger sporogenic nuclei (sn) **G** the nuclei of thin-walled young spores are stained **H** in mature sporogenic plasmodia, only the vegetative nuclei between the spores are stained (arrows) **I, J** flattened mature spores with central spore opening on one side (**I**) **K** ultra-thin section of a sporogenic plasmodium with uninucleated mature spores and vegetative nuclei in the mother cytoplasm. n = nuclei **L** thin-walled young spore with a nucleus in polar position, mitochondria (mi) and endoplasmic reticulum (er) **M** maturing spore with attached vesicles (ve) delivering spore wall material and centrally located nucleus **N, O** mature spores with five-layered, thick spore wall, dense cytoplasm and central nucleus. Scale bars: 10 µm (**A–H**); 5 µm (**K**); 1 µm (**I, J, L–O**).

The most obvious sign of an infection with nephridiophagids were the sporogenic plasmodia in the lumens of the Malpighian tubules and the plasmodia and spores released in the squash preparations (Fig. 2A–C). The sporogenic plasmodia contained 7–36 (mean 21.5) spores (n = 21; Fig. 1A, B). Vegetative plasmodia had varying sizes and accordingly few or many nuclei (Fig. 2B, D–F). Giemsa staining revealed single nuclei in young spores (Fig. 2G) and the typical vegetative nuclei in the mother cytoplasm of sporogenic plasmodia (Fig. 2H). The Giemsa stain was not able to penetrate the spore wall of mature spores so that their nuclei remained uncontrasted (Fig. 2H). Mature spores were flattened oval (Fig. 2C, I, J) and featured a small central spore opening on one side (Fig. 2I). They measured 3.6–4.7 (mean 4.2) µm in length and 2.1–2.5 (mean 2.3) µm in width (n = 20). Ultrathin sections revealed more details of the sporulation process. In the sporogenic plasmodia, future spore nuclei and their surrounding cytoplasm were delimited from the mother cytoplasm by the developing spore walls (Fig. 2K–O). Thus, spores were formed inside the plasmodium, while residual vegetative nuclei remained in the mother cytoplasm (Fig. 2K). In the moderately electron-dense cytoplasm of young spores, mitochondria and endoplasmic reticulum could be seen (Fig. 2L). Their single nucleus was either located at the cell pole (Fig. 2L) or in the centre (Fig. 2M). The initially thin, electron-dense spore wall increasingly thickened, especially at its borders (Fig. 2M–O). In mature spores, the spore wall contained five layers (Fig. 2N, O). Layers 1 and 5 were thin and darkly contrasted, while the other three layers were thicker and became brighter inwards.

### ﻿Phylogenetic position of nephridiophagids

**Figure 3.**
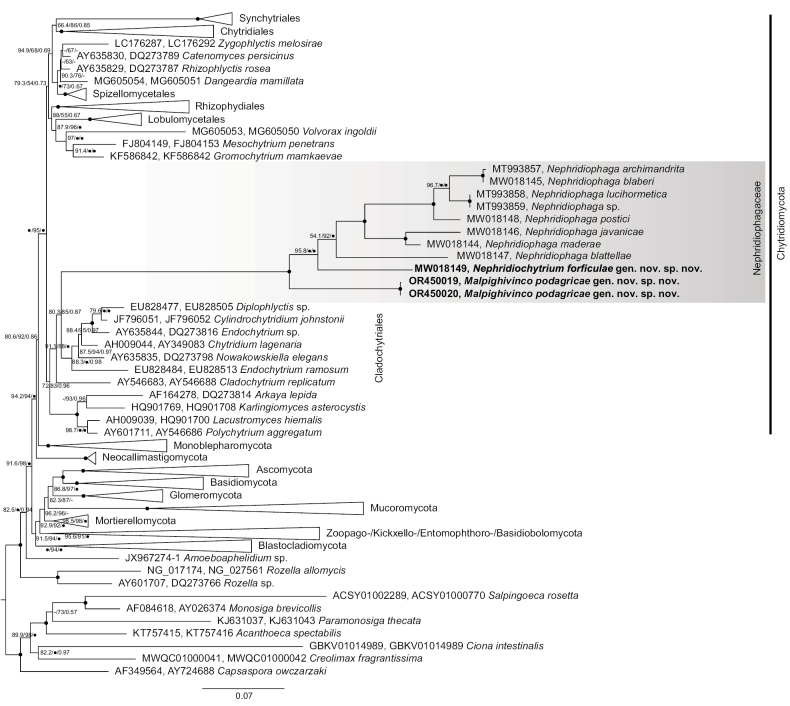
Phylogenetic tree inferred from a concatenated alignment of SSU and LSU rRNA genes under the GTR+F+R5 model. Branch support is given by SH-aLRT/UFBOOT2/Bayesian posterior probabilities. Black circles indicate support values ≥ 99% or ≥ 0.9 and dashes indicate values < 50% or < 0.5. Black circles at branches show ≥ 99% and ≥ 0.9 support in all analyses. Sequences of the here newly-described species are marked in bold. For the nephridiophagids obtained from the two *Lucihormetica* species and from *Archimandritatessellata*, only the SSU rRNA gene sequence was available for tree inference.

Phylogenetic analyses of a concatenated SSU and LSU rRNA gene sequence alignment of the newly-described nephridiophagids along with other nephridiophagids and major fungal groups confirmed their affiliation to the Nephridiophagaceae (Fig. 3). Both the sister-relationship of the nephridiophagid from *Forficulaauricularia* to *Nephridiophaga* (from cockroach hosts) as well as the sister-relationship of the nephridiophagid from *Podagricamalvae* to all other nephridiophagids was inferred with maximum support (UFBOOT2 and Bayesian posterior probability). However, whereas the branching of Nephridiophagaceae within the Chytridiomycota (as discovered before; Strassert et al. (2021)) was inferred with confidence, the exact position of the family remained ambiguous. Members of this family were nested within the Cladochytriales, but statistical support for this branching was rather low (Fig. 3). Whether or not the family Nephridiophagaceae is affiliated to the Cladochytriales or represents a distinct order (Nephridiophagales; as proposed by Doweld (2014)) could, therefore, not be resolved here.

### ﻿Infection status of examined insects

All five examined earwigs collected in France were infected, i.e. they all had a high number of parasite stages in the Malpighian tubules. In contrast, only three of 16 earwigs from Germany were positive and their infection intensity was low. In addition to nephridiophagids, some of the German earwigs contained gregarines in the gut and microsporidia in the haemolymph (not shown). With nephridiophagids infected mallow beetles of the genus *Podagrica* could only be found on Sicily, Italy (3 of 14 beetles from two collection sites). Since some of the beetles were already dead at the time point of dissection, the origin and identity of similar-sized spores in the bodies of two further beetles remained unclear. We identified spores in the gut of two more beetles as microsporidia by transmission electron microscopy (not shown). None of the 30 beetles collected in Germany contained nephridiophagids. Occasionally, gregarines were found in the guts of the Italian beetles (not shown). Generally, the insects from Germany were less or not at all infected, while those collected in the Mediterranean area (France, Italy) showed a higher rate of nephridiophagids.

## ﻿Discussion

In this study, we characterised two non-cockroach nephridiophagids by their morphology and SSU and LSU rRNA gene sequences. The nephridiophagid from *Forficulaauricularia* was initially named by Léger (1909) as *Peltomycesforficulae*, but, except from the information that it is very similar to *Peltomyceshyalinus* from the beetle *Olocratesabbreviatus*, hardly any further data were given. Later, the pathogen was investigated closer by light microscopy and assigned to the genus *Nephridiophaga* (*Nephridiophagaforficulae*; Ormières and Manier (1973)). According to Léger (1909), the spores measured 6.4 × 3.3 µm and, according to Ormières and Manier (1973), their sizes were 5 × 2.5 µm (stained) or 7 × 4 µm (fresh). Since these size ranges match well with our measurements of fresh spores (6.3 × 3.2 μm), we assume that we found the same pathogen species in *F.auricularia* as those authors described before. The here-presented light and electron microscopic results showed that the morphology and life cycle stages of the pathogen closely resemble *N.forficulae* and other described nephridiophagids. There were no major differences to the genera *Peltomyces* or *Nephridiophaga*. An assignment to the genera *Oryctospora* and *Coleospora* could be excluded since mature spores possessed neither “alae” (wing-like extensions), such as in *Oryctospora* nor two nuclei, such as in *Coleospora*. Spore size and number of produced spores within the sporogenic plasmodium differ within members of individual genera and, thus, do not allow us to differentiate them (Radek and Herth 1999). The layers of the spore walls of different nephridiophagid species seem to vary slightly in structure and number (Toguebaye et al. 1986; Purrini and Weiser 1990; Lange 1993; Radek and Herth 1999; Fabel et al. 2000; Radek et al. 2002). However, these differences do not allow distinction and may be influenced by, for example, fixation and contrasting conditions. The only but decisive feature justifying the erection of a new genus for the nephridiophagid from *F.auricularia* is molecular data. Our phylogenetic analyses clearly show that the earwig parasite is sister to a cluster of *Nephridiophaga* species from cockroaches. We do not retain the genus name *Peltomyces* since the type species *P.hyalinus* is from a beetle host and it does not seem to be likely that these two pathogens belong to the same genus. We, therefore, propose the new genus name *Nephridiochytrium* for the earwig pathogen and describe the new species as *Nephridiochytriumforficulae*.

Nephridiophagids have already been found in some beetles, including a mallow beetle of the genus *Podagrica* (Léger 1909; Gibbs 1959; Toguebaye et al. 1986; Purrini and Rhode 1988; Purrini and Weiser 1990). Purrini and Rhode (1988) described an infection in *Podagricafuscicornis* and classified it as *Coelosporidiumschalleri*, presuming that the spore-former belongs to the Haplosporidia. However, the ultrastructure of the type species of the genus *Coelosporidium*, *Coelosporidiumchydoricola* Mesnil & Marchoux, 1897, clearly differs from that of nephridiophagids (Manier et al. 1976). An affiliation to the Haploridia was therefore excluded and the pathogen was transferred to the genus *Nephridiophaga* as *Nephridiophagaschalleri* (Lange 1993). The nephridiophagid from the mallow beetle *Podagricamalvae* has similar life cycle stages as the one from *P.fuscicornis*. The spore form of *N.schalleri*, however, is described as cylindrical with round poles (Purrini and Rhode 1988), while our electron microscopic studies of the nephridiophagid from *P.malvae* and from many cockroach *Nephridiophaga* species (e.g. Radek and Herth (1999); Fabel et al. (2000); Radek et al. (2017)) showed a flattened oval form. Presumably, the flattening of the spores has not been recognised in the light microscopic investigations by Purrini and Rhode (1988). However, there are other differences between our newly-described nephridiophagid and *N.schalleri*. The sporogenic plasmodia of the new nephridiophagid from *P.malvae* contained considerably fewer (7–36 instead of 24–64) and shorter spores (3.6–4.7 × 2.1–2.5 µm instead of 4.5–5 × 2.0–2.5 µm). Due to these differences and the general host specificity of nephridiophagids (Woolever 1966), we presume the presence of distinct nephridiophagid species in different *Podagrica* species. The nephridiophagids from the two *Podagrica* species show no relevant morphological or life cycle differences to other nephridiophagid genera, except *Coleospora* and *Oryctospora* (see above). In our phylogenetic tree, however, the sequence from *P.malvae* was the most divergent nephridiophagid, branching apart from all other nephridiophagids from cockroaches and the earwig. Considering the need for a distinguishing genus name for this parasite, we propose the new genus name *Malpighivinco* and name the new species *Malpighivincopodagricae*.

An unusual common feature of all nephridiophagids is the presence of two types of nuclei in the sporogenic plasmodia. The larger nuclei are the future spore nuclei, while the smaller ones remain in the mother cytoplasm. The presence of two nucleus types in the context of spore formation is reminiscent of stages found in Aphelidiomycota (Tcvetkova et al. 2023) — a sister group to true fungi (Torruella et al. 2018; Strassert and Monaghan 2022) with life cycles resembling those of Chytridiomycota. The stage with two nucleus types is represented by a multinucleate plasmodium of unclear origin. While division releases zoospores inheriting one nucleus type, the remnant of the plasmodium (‘monster’) retains the other nucleus type and seems to degenerate after a short motile phase (Tcvetkova et al. 2023). The presence of two nucleus types in the life cycle may be an ancient trait of (early-diverging) fungi and their fungus-like aphelid relatives.

Our study shows that all known nephridiophagid species cluster together as a well-supported monophyletic lineage within the Chytridiomycota. Moreover, the distinct clustering of the two new genera together with *Nephridiophaga* support the presumption that the nephridiophagids, similar to other parasites, evolved in parallel with their hosts. A broader host screening may help to shed light on the origin and host range of nephridiophagids. Currently, their closer affiliation remains enigmatic and whether or not nephridiophagids form a distinct order (Nephridiophagales) as supposed by Doweld (2014) or belong to the Cladochytriales will have to be scrutinised once more sequence data become available.

## ﻿*Taxonomy*

### ﻿Opisthokonta, Fungi, Chytridiomycota, Nephridiophagales, Nephridiophagaceae

#### ﻿ Nephridiochytrium

Taxon classificationFungiNephridiophagalesNephridiophagaceae

Radek & Strassert gen. nov.

1A6F5643-A671-57F2-8634-6C17ADC3B76F

 850000

##### Etymology.

“Nephridio” refers to the site of infection, the nephridia, i.e. the insect kidneys (Malpighian tubules) and “chytrium” refers to the fungal assignment within the chytrids: *Nephridiochytrium*.

##### Diagnosis.

Typical life cycle stages and morphology of Nephridiophagaceae.

#### ﻿ Nephridiochytrium forficulae

Taxon classificationFungiNephridiophagalesNephridiophagaceae

Radek & Strassert sp. nov.

BE0EE660-A127-5F33-B8A1-41784C5A1EF2

 850001

##### Etymology.

“forficulae” refers to the host, an earwig of the genus *Forficula*.

##### Diagnosis.

Oval, flattened spores measuring 5.8–6.9 (mean 6.3) × 2.9–3.5 (mean 3.2) μm; 13–37 (mean 19) spores per sporogenic plasmodium; central capped spore-opening at one side of mature spores. Oligo- and multinucleated vegetative plasmodia.

##### Type host.

*Forficulaauricularia* Linnaeus 1758 (Dermaptera, Forficulidae). COII gene accession number MN528021.

##### Type host locality.

Tours, France.

##### Syntype.

Cells in Fig. 1.

##### Gene sequence.

rDNA operon acc. no. MW018149.

#### ﻿ Malpighivinco

Taxon classificationFungiNephridiophagalesNephridiophagaceae

Radek & Strassert gen. nov.

1BBC90AA-7B66-5ED9-8F03-65E40F924493

 850002

##### Etymology.

“Malpighi” refers to the Malpighian tubules as habitat and the Latin word “vinco” (verb “vincere”) means “I conquer”, i.e. the pathogens infect the Malpighian tubules: *Malpighivinco*.

##### Diagnosis.

Typical life cycle stages and morphology of Nephridiophagaceae.

#### ﻿ Malpighivinco podagricae

Taxon classificationFungiNephridiophagalesNephridiophagaceae

Radek & Strassert sp. nov.

9839F90C-5830-512F-9636-3BD149B8A39A

 850003

##### Etymology.

“podagricae” refers to the host, a mallow beetle of the genus *Podagrica*.

##### Diagnosis.

Oval, flattened spores measuring 3.6–4.7 (mean 4.2) × 2.1–2.5 (mean 2.3) µm; 7–36 (mean 21.5) spores per sporogenic plasmodium; central capped spore-opening at one side of mature spores. Oligo- and multinucleated vegetative plasmodia.

##### Type host.

The mallow beetle *Podagricamalvae* (Illiger 1807).

##### Type host locality.

Sicily, Italy.

##### Syntype.

Cells in Fig. 2.

##### Gene sequence.

rDNA operon acc. no. OR450019 and OR450020.

## ﻿Conclusions

The here-described two new genera of Nephridiophagaceae show only few morphological and life cycle differences to other members of the family. A lack of distinctive morphological features certainly is a result of adaptations to the parasitic life style. All yet detected members of nephridiophagids live in the same habitat, namely the Malpighian tubules of insects. Thus, development of special structures or new multiplication strategies were seemingly not necessary to survive in different host taxa. Nevertheless, distinct phylogenetic lineages evolved in different insect taxa. The genus *Nephridiophaga*, for example, seems to be restricted to cockroach hosts, while beetles and earwigs are infected by other nephridiophagid genera.

## Supplementary Material

XML Treatment for Nephridiochytrium

XML Treatment for Nephridiochytrium forficulae

XML Treatment for Malpighivinco

XML Treatment for Malpighivinco podagricae

## ﻿Additional information

### Author ORCIDs

Renate Radek https://orcid.org/0000-0001-7605-7546

Christian Wurzbacher https://orcid.org/0000-0001-7418-0831

Jürgen F. H. Strassert https://orcid.org/0000-0001-6786-7563

### Data availability

All generated sequences were submitted to GenBank.

## Supplementary materials

Supplementary material 1 Infections of *Podagrica* spp. and *Forficulaauricularia* with nephridiophagids (in Malpighian tubules) and other pathogens Data type xlsx Explanation note The table gives an overview on the infection status of earwigs and mallow beetle collected at different locations. https://binary.pensoft.net/file/956742This dataset is made available under the Open Database License (http://opendatacommons.org/licenses/odbl/1.0/). The Open Database License (ODbL) is a license agreement intended to allow users to freely share, modify, and use this Dataset while maintaining this same freedom for others, provided that the original source and author(s) are credited.Renate Radek

Supplementary material 2 SSU rRNA gene sequence alignments used for phylogenetic tree reconstruction (Figure 3) Data type fa https://binary.pensoft.net/file/956743This dataset is made available under the Open Database License (http://opendatacommons.org/licenses/odbl/1.0/). The Open Database License (ODbL) is a license agreement intended to allow users to freely share, modify, and use this Dataset while maintaining this same freedom for others, provided that the original source and author(s) are credited.Jürgen F. H. Strassert

Supplementary material 3 LSU rRNA gene sequence alignments used for phylogenetic tree reconstruction (Figure 3) Data type fa https://binary.pensoft.net/file/956744This dataset is made available under the Open Database License (http://opendatacommons.org/licenses/odbl/1.0/). The Open Database License (ODbL) is a license agreement intended to allow users to freely share, modify, and use this Dataset while maintaining this same freedom for others, provided that the original source and author(s) are credited.Jürgen F. H. Strassert
